# The Kidney Awareness Registry and Education (KARE) study: protocol of a randomized controlled trial to enhance provider and patient engagement with chronic kidney disease

**DOI:** 10.1186/s12882-015-0168-4

**Published:** 2015-10-22

**Authors:** Delphine S. Tuot, Alexandra Velasquez, Charles E. McCulloch, Tanushree Banerjee, Yunnuo Zhu, Chi-yuan Hsu, Margaret Handley, Dean Schillinger, Neil R. Powe

**Affiliations:** Division of Nephrology, University of California, San Francisco, San Francisco, CA 94143 USA; Division of General Internal Medicine at San Francisco General Hospital, University of California, San Francisco, San Francisco, CA 94143 USA; Department of Epidemiology and Biostatistics, University of California, San Francisco, San Francisco, CA 94143 USA; Center for Vulnerable Populations at San Francisco General Hospital, San Francisco, CA USA; Renal Center at San Francisco General Hospital, 1001 Potrero Avenue Bldg 100, Room 342, San Francisco, CA 94110 USA

**Keywords:** Chronic kidney disease, Self-management, CKD awareness, Health coaching

## Abstract

**Background:**

Chronic kidney disease (CKD) is common and is associated with excess mortality and morbidity. Better management could slow progression of disease, prevent metabolic complications, and reduce cardiovascular outcomes. Low patient awareness of CKD and ineffective patient-provider communication can impede such efforts. We developed provider and patient-directed interventions that harness health information technology to enhance provider recognition of CKD and delivery of guideline concordant care and augment patient understanding and engagement in CKD care.

**Methods/design:**

We report the design and protocol of the Kidney Awareness Registry and Education (KARE) Study, a 2x2 factorial randomized controlled trial that examines the impact of a multi-level intervention on health outcomes among low-income English, Spanish and Cantonese-speaking patients with CKD in a safety net system. The intervention includes: (1) implementation of a primary care electronic CKD registry that notifies practice teams of patients’ CKD status and employs a patient profile and quarterly feedback to encourage provision of guideline-concordant care at point-of-care and via outreach; and (2) a language-concordant, culturally-sensitive self-management support program that consists of automated telephone modules, provision of low-literacy written patient-educational materials and telephone health coaching. The primary outcomes of the trial are changes in systolic blood pressure (BP) and the proportion of patients with BP control (≤140/90 mmHg) after one year. Secondary outcomes include patient understanding of CKD, participation in healthy behaviors, and practice team delivery of guideline-concordant CKD care.

**Discussion:**

Results from the KARE study will provide data on the feasibility, effectiveness, and acceptability of technology-based interventions that support primary care efforts at improving health outcomes among vulnerable patients with CKD.

**Trial registration:**

ClinicalTrials.gov, number: NCT01530958

## Background

Chronic kidney disease (CKD) is common, with an estimated prevalence of 11.5 % among the U.S. adult population [1], causes excess mortality [2], and is associated with significant socio-demographic disparities [3, 4]. Racial/ethnic minorities and the poor, often treated in safety-net health systems, are more likely to have CKD at an earlier age [5–7]. Although randomized controlled trials have demonstrated that measures such as blood pressure control [8], reduction of proteinuria with angiotensin converting enzyme inhibitors (ACEi) or angiotensinogen receptor blockers (ARB) [9–11], and glycemic control among persons with diabetes [12, 13], can delay CKD decline and decrease CKD-associated morbidity and mortality [14], many individuals with CKD are not benefiting from these scientific advances. Lack of translation may be due to low levels of CKD awareness among providers and patients [15–17]; low self-efficacy among primary care providers for delivery of CKD care [18], particularly in an inefficient health care system with overburdened providers that deliver chronic disease care; and poor patient empowerment to participate in healthy lifestyles, adhere to medication regimens, and avoid nephrotoxic insults [19, 20].

The Chronic Care Model posits that an informed patient and prepared practice team have productive interactions that lead to improved outcomes [21]. It provides a framework for the delivery of high-quality chronic disease care and can be integrated into the Patient Centered Medical Home [22]. Implementation of single elements of the Chronic Care Model (e.g., health care organization, community resources, patient self-management support, delivery system re-design, decision support) can improve processes of care, such as decreased hospitalizations among patients with congestive heart failure [23]. Interventions that have enhanced patient outcomes have incorporated several elements of the Chronic Care Model [24]. For example, data from the North Carolina Improving Performance in Practice program, a state-wide quality improvement program aimed at improving health outcomes among patients with diabetes, demonstrated a positive graded association between improved cholesterol levels among patients with diabetes and the extent to which clinical practices implemented and used the following components of the multi-level intervention: diabetes and lipid registry, list of standardized items that are addressed with every diabetic patient at every visit, comprehensive care protocols for diabetes management and patient self-management support systems [25].

**Fig. 1:**
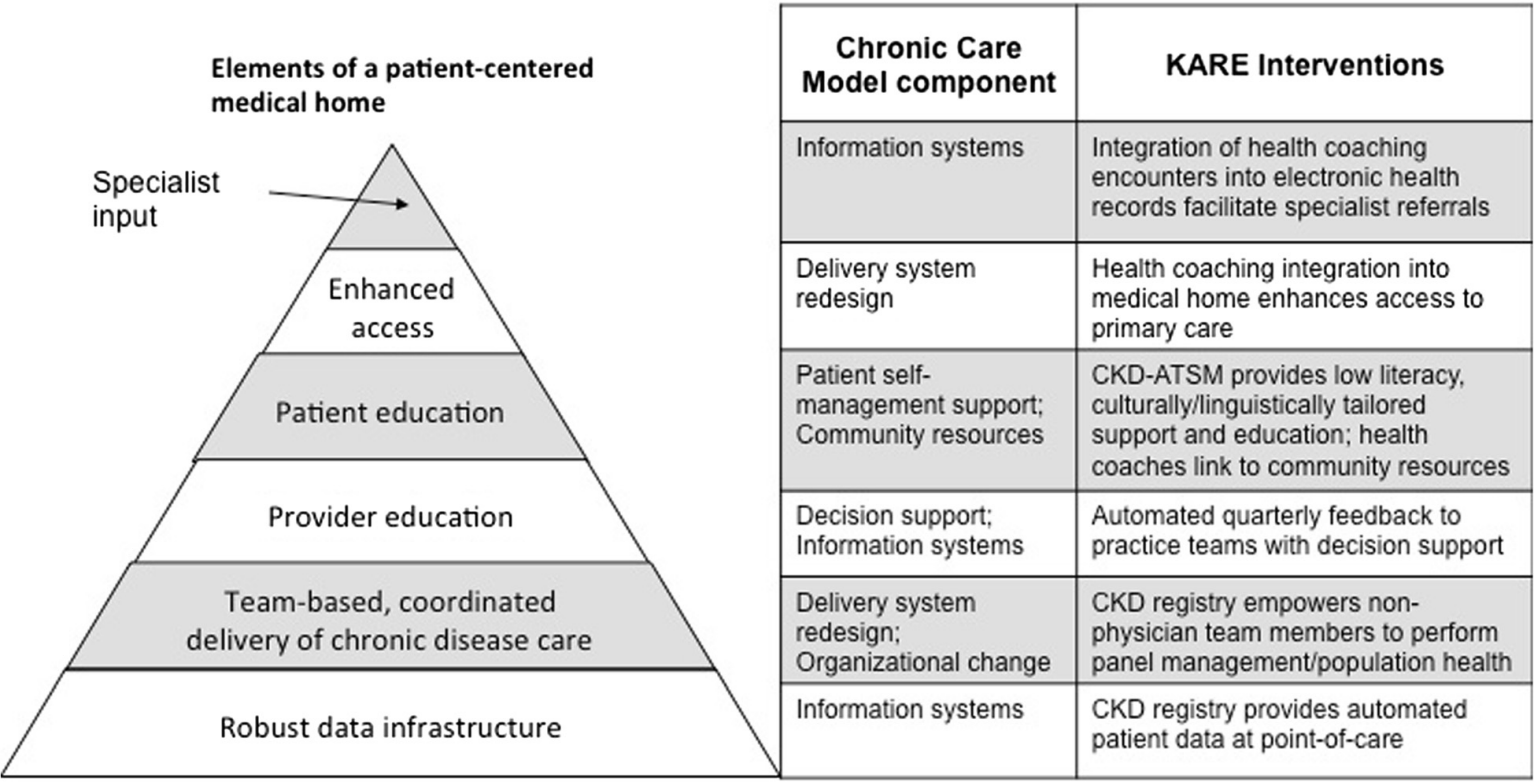
KARE incorporates components of the Chronic Care Model and integrates with a patient-centered medical home to enhance health outcomes

Sustainable multi-level interventions that enhance CKD management in primary care settings are rare, and none have been studied in U.S. safety-net delivery systems, where vulnerable populations (e.g., the poor, minorities, limited health literacy/English proficiency) bear a disproportionate burden of disease [5, 26] and experience large translational gaps between research and practice [27]. We describe the design and protocol of the Kidney Awareness Registry and Education (KARE) study, which assesses the effectiveness and feasibility of a multi-level intervention to improve blood pressure control among low-income patients with CKD. The intervention targets primary care practice teams and patients using multiple elements of the Chronic Care Model, including delivery system redesign, decision support, patient self-management support, organizational change and information systems. It also harnesses the core elements of a Patient Centered Medical Home, including robust data infrastructure, team-based delivery of chronic disease care and provider and patient education. The KARE intervention consists of: (1) an electronic CKD registry that provides point-of-care decision support to clinicians regarding CKD management; (2) outside-of care summaries of CKD relevant clinical data to optimize delivery of guideline-concordant CKD care by practice teams; and (3) a comprehensive patient self-management support program, entitled CKD-ATSM (automated telephone self-management), that includes low-literacy educational materials; proactive, automated telephone self-management support; and live telephone health coaching (Fig. 1).

## Methods/design

### Study design

**Fig. 2:**
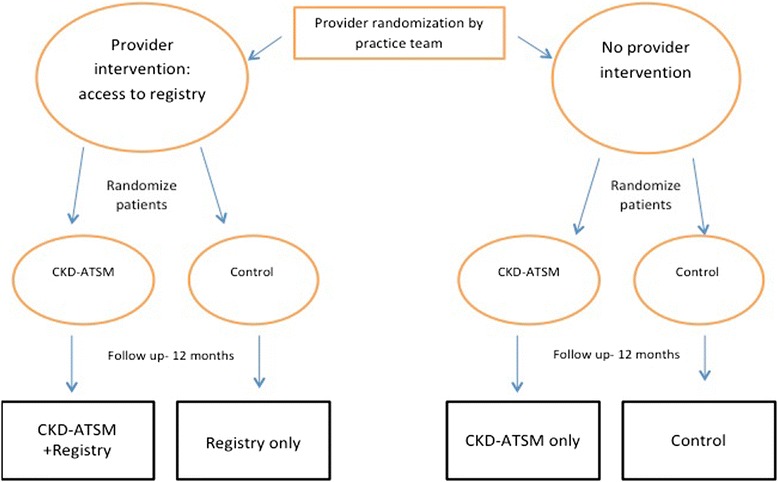
KARE 2x2 factorial clinical randomized trial design

KARE is a non-blinded 2x2 factorial randomized controlled trial with two levels of randomization. Primary care providers are randomized to one of two arms: access to an electronic CKD registry or usual care. Patients are subsequently randomized to one of two arms: CKD self-management program (CKD-ATSM) or usual care. This study design, with 4 study arms, allows assessment of the individual and additive impact of both interventions (Fig. 2). Approval to conduct this study was granted by the Committee of Human Research at the University of California, San Francisco.

### Study population and setting

Patients with CKD and their providers are recruited from primary care clinics in the San Francisco Health Network, the integrated public health care delivery system serving San Francisco’s uninsured and publicly insured residents. The study team collaborates with the staff at each clinic to implement and monitor the study.

### Eligibility criteria

#### Providers

All primary care providers, including attending physicians, family practice and internal medicine trainees, nurse practitioners and physician assistants who provide longitudinal primary care to patients, are eligible for this study. Providers who solely provide specialty care, for example HIV services, psychiatric care or urgent care, are excluded from the study.

#### Patients

Eligible patients include adults (>18 years) with CKD, defined by an eGFR 15–60 ml/min/1.73 m^2^ or presence of at least two 1+ dipstick albuminuria separated by at least 90 days. Estimated GFR is calculated using the Modified Diet in Renal Disease study equation [28], as this is automatically reported by the electronic medical record (EMR). Patients must have had contact with their primary health care team at least once within the past 2 years and speak English, Spanish or Cantonese. Kidney transplant recipients, pregnant women, and individuals on dialysis are excluded from this study. Other exclusion criteria include those that impede meaningful communication between providers and patients or limit the usefulness of a CKD self-management support program: prevalent dementia, impaired cognition or severe mental illness; expected life expectancy <6 months; self-reported hearing impairment or severe visual impairment preventing use of a touchtone telephone keypad.

### Identification and recruitment

#### Providers

Clinical leadership of primary care clinics in the San Francisco Health Network are approached to participate in this study. All providers from participating clinics are recruited to take part in KARE.

#### Patients

The San Francisco Health Network’s electronic patient registry is searched to identify eligible study participants. Primary care providers are sent a letter outlining the study objectives with a list of their potentially eligible patients. Providers are asked to identify patients who should be excluded from the study based on the aforementioned exclusion criteria. After receiving provider approval, eligible patients are contacted via telephone to inform them of the study, ensure eligibility, and schedule an enrollment appointment. Voicemails are left for patients who do not respond. For patients who do not respond to 3 phone calls or messages, an attempt is made to call the emergency contact individual listed in the EMR. Successful contact results in an update of the eligible patient’s contact information in the EMR and three additional attempts to contact the patient.

### Enrollment and randomization

#### Providers

Consistent with the patient-centered medical home, clinicians in participating primary care clinics are assigned to practice teams responsible for distinct panels of patients. Each team consists of several physicians, nurses, nurse practitioners, medical assistants and behaviorists. Within each clinic, patient demographics across practice teams are similar with respect to race/ethnicity, gender, age and language distribution. To minimize contamination among clinicians, practice teams, rather than primary care providers, are randomized to receive the CKD registry or to receive usual care.

#### Patients

Prior to enrollment, using blind and secure allocation by computer, the study team randomly assigns participants to one study arm using a random number generator: CKD-ATSM or usual care. Randomization is blocked and stratified within primary care provider to increase the comparability of treatment groups and to ensure equal allocation by provider. Results are placed in a sealed randomization envelope by a study coordinator.

At the study enrollment appointment, the study team obtains written informed consent and collects baseline study measures. At the completion of this baseline visit, patients learn about their randomization assignment by opening the randomization envelope. Subjects randomized to the intervention arm are provided printed instructions about how to participate in the telephone self-management program and are guided through the process of creating a personalized log-in code for the automated telephone modules. Patients are also given a business card from his/her assigned health coach and a schedule of study events. If randomized to usual care, patients only receive a schedule of study events. All patient participants receive a $50 gift card to a local supermarket for their participation in the study.

### Interventions

#### Provider intervention

The CKD registry was designed to alert clinicians of a patient’s CKD-relevant information and was created with input from San Francisco Health Network clinical leadership and quality improvement champions [29]. This registry, which uses i2iTracks software (http://www.i2isys.com/p/i2itracks), searches the EMR and identifies individuals with CKD based on eGFR and albuminuria, either by dipstick or urinary albumin:creatinine ratio. A printed patient profile provides the primary care practice team with point-of-care data on CKD status, recent blood pressure readings, prescription of pertinent medications (aspirin, ACEi, ARB, statin), recent quantification of albuminuria and recent low-density lipoprotein values, as well as non-CKD specific data that practice teams in the control group receive: immunization status and data pertinent to age appropriate cancer screening. Practice team medical assistants use the patient profile while obtaining vital signs during the clinic visit to identify all patients who are not up to date with their age appropriate cancer screening and patients with CKD who need albuminuria quantification and immunizations. The registry also encourages them to notify primary care providers about lapses in guideline-concordant CKD care delivery by highlighting blood pressure readings that are above 140/90 mmHg and noting when patients are not on an ACEi or ARB. Written decision support on the patient profile also reminds primary care providers to avoid prescribing non-steroidal anti-inflammatory medications for patients with CKD and to prescribe statin medications when appropriate.

Quarterly written feedback to practice teams focuses on three important groups of  patients with CKD: those with uncontrolled BP, those not prescribed an ACEi or ARB, and those with persistent macroalbuminuria [14]. Individual PCPs also receive printed sheets identifying their patients who fall in the above categories.

#### Provider usual care

Usual care consists of a patient profile that only includes data pertinent to age-appropriate cancer screening. Practice team medical assistants use the patient profile while obtaining vital signs to identify patients who are not up to date with respect to colon cancer, breast cancer and/or cervical cancer screening.

#### Patient intervention

**Fig. 3:**
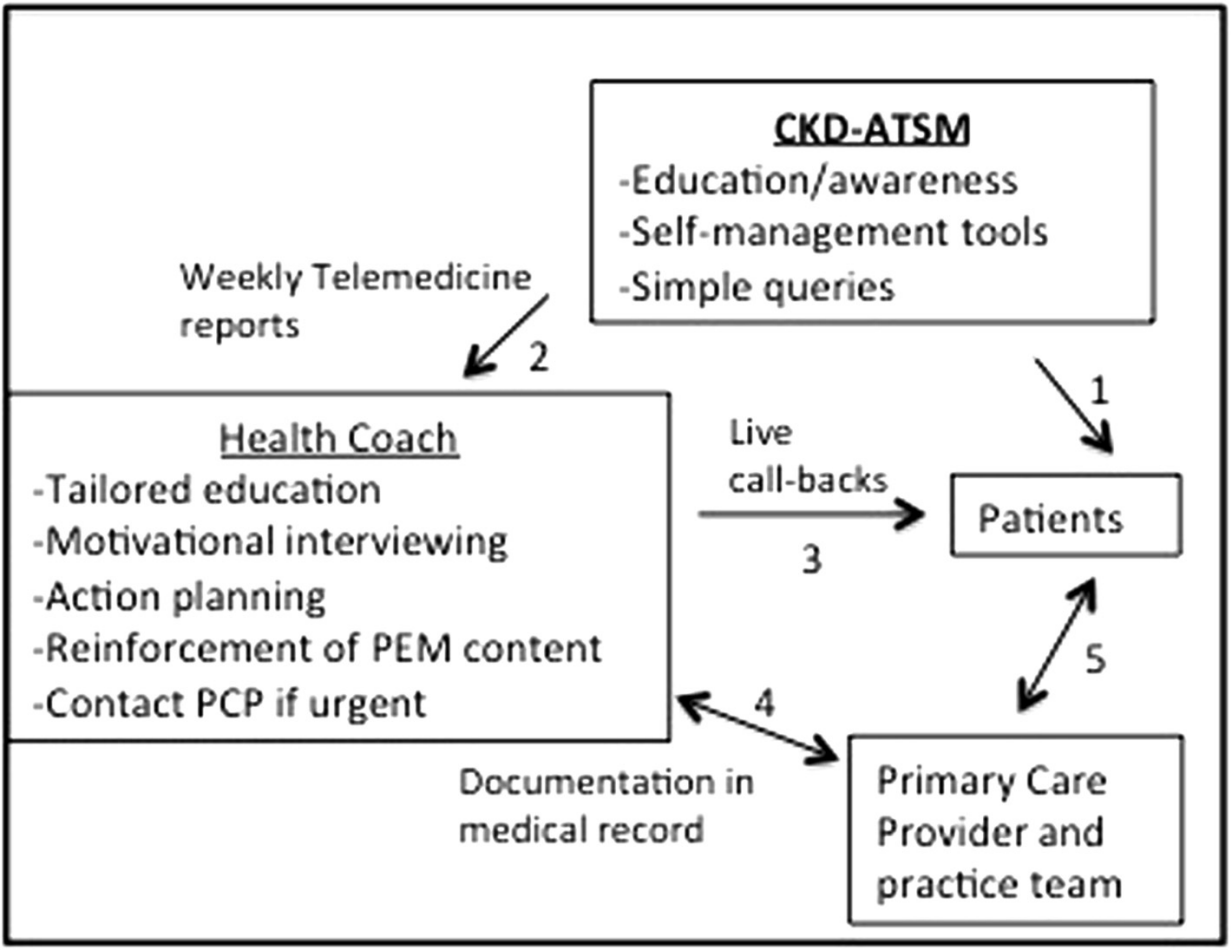
CKD-ATSM blends health technology with personalized education and counseling

The comprehensive CKD self-management support program consists of three distinct elements that are based on the Social Cognitive Theory constructs of behavioral capability, self-efficacy, expectations and reinforcement [30, 31]: (1) patient educational materials about CKD; (2) automated telephone self-management (CKD-ATSM); and (3) live telephone-based health coaching. CKD-ATSM was developed with key input from local clinicians to meet the mutli-lingual, low-literacy and diverse cultural needs of the San Francisco safety-net patient population and has been successfully employed in diabetes care [32]. The program consists of 27 different modules that cover topics pertinent to kidney health: basics of kidney disease and its association with hypertension; importance of participation in healthy behaviors (diet, physical activity, smoking cessation, stress reduction); avoidance of non-steroidal anti-inflammatory medications; participation and preparation for clinic visits, complementary medication use; medication adherence; and glycemic control. Offered in English, Spanish, and Cantonese, each phone call has educational messages about 1–2 topics (i.e., nephrotoxic medications), followed by culturally tailored vignettes that highlight self-management behaviors that encourage patients to overcome barriers to engage in their own health and decrease risk of morbidity (i.e., use of Acetaminophen instead of NSAIDS to minimize risk of acute kidney injury). Embedded within these automated phone calls are simple queries that allow the study team to capture data and the health coach to respond to out of range values. The study health coaches receive these data on a weekly basis, allowing them to generate live phone calls to those patients who provide alarming or “out or range” data (i.e., responding “3 or greater” to the question “In the last 7 days, how many days did you miss taking a medication?”). During the live phone calls, health coaches can provide additional education and encourage behavior change through motivational interviewing and action planning. Phone interactions are documented in the EMR, providing seamless integration into medical care (Fig. 3).

The bilingual health coaches employed for this study do not have a medical background. To prepare for the KARE study, they attend 18 h of training led by the UCSF Center for Excellence in Primary Care (http://cepc.ucsf.edu/health-coaching). Training is conducted in English, using a curriculum developed by the trainers and study team. Curriculum modules include: working collaboratively with patients; basics of CKD; knowledge of common cardiovascular medications; recognizing “red flags” such as symptoms of depression and not taking any medications; difficulty navigating the clinic; and accessing community resources. Furthermore, coaches are trained to interact with patients using active listening, provide social and emotional support, assist with lifestyle changes and facilitate medication understanding and adherence.

Language-concordant, low-literacy written patient educational materials (PEMs) are given to study participants at baseline and throughout the study period. PEMs were selected after a thorough review of available CKD educational materials [33]. Health coaches refer to these PEMs during their live telephone calls to reinforce concepts and encourage further engagement.

#### Patient usual care

Subjects randomized to not receive CKD-ATSM continue to receive medical care at their primary care clinic. They are eligible to receive any of the clinic’s usual resources, including interactions with non-physician health care providers, such as nurses, pharmacists, and educators. These resources are also available to study participants randomized to receive CKD-ATSM.

### Data collection, follow-up and outcomes

#### Data collection

In addition to clinical and behavioral outcomes, baseline data are collected to describe the characteristics of study participants (providers and patients) and compare these characteristics between intervention groups assigned by randomization. These data include: self-reported provider demographic data (age, gender, years of experience, specialty, preferred language), provider perception of practice team cohesiveness, self-reported patient socio-demographic data (age, gender, race/ethnicity, social support, education, income, health literacy, zip code, insurance status), co-morbid conditions (diabetes, hypertension, cardiovascular disease), food insecurity, and health literacy (Table 1).

**Table 1:** Kidney Awareness Registry and Education (KARE) study variables and outcomes

KARE Study data collection	Method of ascertainment	Baseline	Mid-point	Study end
Primary care providers				
*Demographic data*				
Age, gender, years of experience, language	Survey	+		
*Behavioral data*				
Awareness/ability to identify CKD	questionnaire [35]	+		+
Self-efficacy towards CKD management	questionnaire	+		+
Comfort communicating with patients about CKD	Communication Assessment Tool [36]	+		+
Prescription of ACEi or ARB, albuminuria quantification, nephrology referral	Medical record	+		+
Patients				
*Demographic data*				
Age, gender, race/ethnicity, social support, income, education, zip code, preferred language, insurance status	Survey	+		
Food insecurity, health literacy	Screening questions	+		
*Clinical data*				
BP, Height, Weight, UACR,	Measured by study team	+		+
eGFR, metabolic bone disease parameters, glycosylated hemoglobin	Medical record	+		+
Co-morbid conditions	Medical record	+		+
Referrals, emergency department visits, hospitalizations	Medical record	+		+
Current medications	Medical record and study team	+	+	+
Quality of life, functional status	SF-12 instrument [37]	+		+
*Behavioral data*				
CKD awareness	Survey	+	+	+
Participation in healthy behaviors	BRFSS questionnaire [40]	+	+	+
Self-efficacy for self-management	Stanford self-efficacy tool [39]	+	+	+
Medication Adherence	Morisky Medication Adherence Scale [38]	+	+	+
Communication with providers	Lorig Communication tool [39]	+	+	+

CKD chronic kidney disease, *ACEi* angiotensin converting enzyme inhibitor, *ARB* angiotensin receptor blocker, *UACR* urine albumin:creaitnine ratio, *eGFR* estimated glomerular filtration rate, *QOL* quality of life, *BRFSS* Behavioral Risk Factor Surveillance System

#### Clinical outcomes

The primary outcomes are changes in systolic BP and proportion of patients with BP control (<140/90 mmHg) after one year. The study team will measure BP using an Omron digital blood pressure monitor model HEM-907X during baseline and 12-month study visits. Per study protocol and American Heart Association guidelines, study personnel will obtain 3 BP measures in the right arm after the participant has been sitting quietly for 5 min [34]. An average of the 3 measures is recorded. Secondary clinical outcomes include: changes in urine albuminuria and weight obtained during by the study team, as well changes in eGFR, laboratory measures of metabolic bone disease (serum calcium, phosphorous, 25-OH Vitamin D and parathyroid hormone) and glycosylated hemoglobin among participants with diabetes, all ascertained through the EMR.

#### Behavioral outcomes

Provider and patient behavioral outcomes will inform mechanisms by which the interventions are or are not successful. We will examine changes in provider awareness/identification of CKD, self-efficacy of delivering CKD guideline-concordant care, comfort in provider communication with patients about CKD, prescription of ACEi or ARB, quantification of albuminuria and nephrology referral. These behavioral data will be ascertained at baseline and at 12 months, using validated instruments when available [35, 36] (Table 1). We will also assess changes in patient awareness of CKD, self-efficacy toward chronic disease self-management, participation in healthy behaviors, medication adherence, and comfort in communicating with providers [37–40] (Table 1).

#### Process measures

Many interventions with efficacy ultimately fail because they are not sustained in clinical practice. KARE interventions were designed for integration into clinical care. We will use the Reach, Effectiveness, Adoption, Implementation, and Maintenance (RE-AIM) framework of program evaluation and implementation to evaluate process and feasibility measures [41]. To examine adoption of KARE interventions, we will survey clinicians and primary care clinic staff about integration with clinic workflow. We will also assess implementation or fidelity of the self-management program with data from the ATSM information technology platform and audio recordings of health coach conversations. To assess maintenance or sustainability of KARE interventions, we will ask patients about overall satisfaction, comprehension of self-management support, and burden of the intervention during language-concordant focus groups. Provider and practice team satisfaction with the program will be ascertained via survey and focus groups respectively. The resources associated with starting and maintaining each successful intervention will be calculated including personnel time, equipment, and information technology (Table 2).

**Table 2:** Process outcomes to be studied in the Kidney Awareness Registry and Education (KARE) study

RE-AIM dimension	Outcome	Method of Assessment
Adoption	1. Are providers and staff satisfied with integration into clinic work flow?	Survey
Implementation	1. Are automated telephone recordings delivered as intended?	Data from ATSM information technology platform; comparison of audio recordings of health coach conversations vs. CKD-ATSM manual
	2. Is the health coaching intervention delivered as intended?	
	3. Is health coaching similar across all health coaches, despite language differences?	
Maintenance (patient)	1. Are there any difficulties in participating? Unanticipated issues?	Focus groups and questionnaire at study conclusion
	2. Are there any unintended consequences of receiving self-management support?	
Maintenance (Provider)	1. Do providers read the electronic notes entered by the health coach?	Focus groups and questionnaire at study conclusion
	2. Do medical assistants use the patient profile routinely?	
	3. Are there unanticipated consequences of the CKD registry on delivery of non-CKD chronic disease care?	
Maintenance	1. Time needed by practice teams to engage with CKD-registry and perform outreach	Health Coach logs, study reports
	2. Costs (supplies, time spent by health coaches and their salaries) for delivery of CKD-ATSM	

*CKD* chronic kidney disease, *ATSM* automated telephone self-management

### Sample size considerations

Sample size and power calculations were performed for the main outcome of interest – change in systolic BP (sBP) after 1 year due to each intervention individually – using effects sizes and standard deviations from multiple published trials of interventions to improve BP control. Baseline data among patients with CKD in the San Francisco Health Network demonstrate an average sBP of 151 mmHg (range: 140–160) across primary care providers, with an intra-class coefficient = 0.0008. Thus, we used simple *t*-test calculations to determine power analysis. Using a two-tailed alpha of 0.05 and beta of 0.8, we need to enroll 200 patients to each intervention (CKD-ATSM vs. usual care and CKD-registry vs. usual care) to detect a 6-point change in systolic BP. This is similar to that achieved by previous team-based intervention trials [42]. Using an interaction analysis, we will also be able to detect a 12-point difference in systolic BP between the arm that receives no intervention and the arm that receives both interventions. To account for a 10 % loss to follow-up, we will need to enroll 220 patients to each intervention, or 110 patients to each arm of the 2x2 factorial design.

### Data analysis

Evaluation of the individual effectiveness of each intervention, as well as their additive impact, will be performed with intention to treat analyses using generalized estimating equations adjusting for patient age, clinic and preferred language, with robust standard errors to account for clustering by provider and repeated patient measures within provider. If significant differences in baseline characteristics (i.e., age, diabetes status) are found among subjects in each study arm, analyses will be adjusted for these differences using multi-variable linear regression. Additional analyses will look for evidence of effect modification by primary language and CKD severity.

## Discussion

Despite scientific advances that can delay CKD decline and decrease CKD-associated morbidity and mortality, and incorporation of these advances into national [43] and international [44] guidelines, delivery of guideline concordant CKD care remains elusive for many kidney disease patients [45, 46]. This is particularly true among patients who receive care in safety-net settings, where translation of evidence into practice is often impeded by low patient awareness of CKD, poor patient engagement in care, competing clinical demands, and an inefficient health care delivery system [47].

Patient self-management support is an important component of chronic care management, yet many primary care practices do not consistently provide this support due to limitations of training, time, and resources. Linguistically and culturally concordant health coaches are an untapped resource to provide this support, as demonstrated by a recent study that demonstrated improved BP, glycemic and lipid control among low-income, hypertensive, diabetic or hyperlipidemic patients working with medical assistant health coaches [48]. The KARE study tests the impact of a comprehensive CKD self-management support program that occurs outside of the primary care encounter and employs accessible technology and lay health coaches to enhance patient awareness of CKD and engagement with care.

Supporting providers in their delivery of CKD-care is key to maximizing the clinical benefits of patient health coaching. The KARE study will test the effectiveness of a primary care electronic CKD registry with elements of point-of-care decision support and out-of-care population health, to empower non-physician practice team members to participate in the delivery of guideline concordant care.

We hypothesize that patients who receive ATSM and health coaching, and whose practice teams are randomized to the registry, will show significant improvement in systolic BP, reduction in albuminuria, and achieve higher self-efficacy for CKD self-management. If KARE interventions are indeed effective at improving health outcomes among safety-net patients with CKD, the identification of feasible and sustainable characteristics will facilitate their dissemination to other delivery systems across the United States, advancing the goal of healthy equity for people with CKD.

## Abbreviations

CKD: Chronic kidney disease
KARE: Kidney Awareness Registry and Education
BP: Blood pressure
ACEi: Angiotensin converting enzyme inhibitor
ARB: Angiotensin receptor blocker
ATSM: Automated telephone self-management
EMR: Electronic medical record
eGFR: Estimated glomerular filtration rate
PEM: Patient educational material

## References

[1] Levey AS, Stevens LA, Schmid CH, Zhang YL, Castro AF, Feldman HI (2009). A new equation to estimate glomerular filtration rate. Ann Intern Med.

[2] Go AS, Chertow GM, Fan D, McCulloch CE, Hsu CY (2004). Chronic kidney disease and the risks of death, cardiovascular events, and hospitalization. N Engl J Med.

[3] Merkin SS, Roux AV, Coresh J, Fried LF, Jackson SA, Powe NR (2007). Individual and neighborhood socioeconomic status and progressive chronic kidney disease in an elderly population: the cardiovascular health study. Soc Sci Med.

[4] McClellan WM, Newsome BB, McClure LA, Howard G, Volkova N, Audhya P (2010). Poverty and racial disparities in kidney disease: the REGARDS study. Am J Nephrol.

[5] Tarver-Carr ME, Powe NR, Eberhardt MS, LaVeist TA, Kington RS, Coresh J (2002). Excess risk of chronic kidney disease among African-American versus white subjects in the United States: a population-based study of potential explanatory factors. J Am Soc Nephrol.

[6] Hall YN, Choi AI, Chertow GM, Bindman AB (2010). Chronic kidney disease in the urban poor. Clin J Am Soc Nephrol.

[7] Hall YN, Rodriguez RA, Boyko EJ, Chertow GM, O’Hare AM (2009). Characteristics of uninsured Americans with chronic kidney disease. J Gen Intern Med.

[8] Tight blood pressure control and risk of macrovascular and microvascular complications in type 2 diabetes: UKPDS 38. UK Prospective Diabetes Study Group. BMJ 1998, 317(7160):703–713. PMC28659 9732337

[9] Brenner BM, Cooper ME, de Zeeuw D, Keane WF, Mitch WE, Parving HH (2001). Effects of losartan on renal and cardiovascular outcomes in patients with type 2 diabetes and nephropathy. N Engl J Med.

[10] Lewis EJ, Hunsicker LG, Clarke WR, Berl T, Pohl MA, Lewis JB (2001). Renoprotective effect of the angiotensin-receptor antagonist irbesartan in patients with nephropathy due to type 2 diabetes. N Engl J Med.

[11] Randomised placebo-controlled trial of effect of ramipril on decline in glomerular filtration rate and risk of terminal renal failure in proteinuric, non-diabetic nephropathy. The GISEN Group (Gruppo Italiano di Studi Epidemiologici in Nefrologia). Lancet 1997, 349(9069):1857–1863. 9217756

[12] The effect of intensive treatment of diabetes on the development and progression of long-term complications in insulin-dependent diabetes mellitus. The Diabetes Control and Complications Trial Research Group. N Engl J Med 1993, 329(14):977–986. 10.1056/NEJM199309303291401 8366922

[13] Intensive blood-glucose control with sulphonylureas or insulin compared with conventional treatment and risk of complications in patients with type 2 diabetes (UKPDS 33). UK Prospective Diabetes Study (UKPDS) Group. Lancet 1998, 352(9131):837–853. 9742976

[14] KDIGO (2012). Clinical practice guideline for the evaluation and management of chronic kidney disease. Summary of recommendation statements. Kidney Int Suppl 2013.

[15] Plantinga LC, Tuot DS, Powe NR (2010). Awareness of chronic kidney disease among patients and providers. Adv Chronic Kidney Dis.

[16] Tuot D, Plantinga L, Judd S, Muntner P, Hsu C, Warnock DG, et al.. Health behaviors, risk factor control and awareness of chronic kidney disease. Am J Nephrol. In press. 2013. 10.1159/000346712 PMC3649001 23392070

[17] Tuot DS, Plantinga LC, Hsu CY, Jordan R, Burrows NR, Hedgeman E (2011). Chronic kidney disease awareness among individuals with clinical markers of kidney dysfunction. Clin J Am Soc Nephrol.

[18] Tahir MA, Dmitrieva O, de Lusignan S, van Vlymen J, Chan T, Golmohamad R (2011). Confidence and quality in managing CKD compared with other cardiovascular diseases and diabetes mellitus: a linked study of questionnaire and routine primary care data. BMC Fam Pract.

[19] Costantini L, Beanlands H, McCay E, Cattran D, Hladunewich M, Francis D (2008). The self-management experience of people with mild to moderate chronic kidney disease. Nephrol Nurs J.

[20] Bodenheimer T, Lorig K, Holman H, Grumbach K (2002). Patient self-management of chronic disease in primary care. JAMA.

[21] Wagner EH, Austin BT, Davis C, Hindmarsh M, Schaefer J, Bonomi A (2001). Improving chronic illness care: translating evidence into action. Health Aff (Millwood).

[22] American College of Physicians (2010). The Patient-Centered Medical Home Neighbod: The Interface of the Patient Centered Medical Home With Specialty/Subspecialty Practices.

[23] Rich MW, Beckham V, Wittenberg C, Leven CL, Freedland KE, Carney RM (1995). A multidisciplinary intervention to prevent the readmission of elderly patients with congestive heart failure. N Engl J Med.

[24] Bodenheimer T, Wagner EH, Grumbach K (2002). Improving primary care for patients with chronic illness: the chronic care model, Part 2. JAMA.

[25] Halladay JR, Dewalt DA, Wise A, Qaqish B, Reiter K, Lee SY (2014). More extensive implementation of the chronic care model is associated with better lipid control in diabetes. J Am Board Fam Med.

[26] Volkova N, McClellan W, Klein M, Flanders D, Kleinbaum D, Soucie JM (2008). Neighborhood poverty and racial differences in ESRD incidence. J Am Soc Nephrol.

[27] Schillinger D (2007). Literacy and health communication: reversing the ‘inverse care law’. Am J Bioeth.

[28] Levey AS, Bosch JP, Lewis JB, Greene T, Rogers N, Roth D (1999). A more accurate method to estimate glomerular filtration rate from serum creatinine: a new prediction equation. Modification of diet in renal disease study group. Ann Intern Med.

[29] McBride D, Dohan D, Handley MA, Powe NR, Tuot DS. Developing a CKD registry in primary care: provider attitudes and input. Am J Kidney Dis. 2013. 10.1053/j.ajkd.2013.10.012 PMC3969392 24295612

[30] Painter JE, Borba CP, Hynes M, Mays D, Glanz K (2008). The use of theory in health behavior research from 2000 to 2005: a systematic review. Ann Behav Med.

[31] Glanz K, Rimer BK, Viswanath K (2008). Health behavior and health education : theory, research, and practice.

[32] Schillinger D, Handley M, Wang F, Hammer H (2009). Effects of self-management support on structure, process, and outcomes among vulnerable patients with diabetes: a three-arm practical clinical trial. Diabetes Care.

[33] Tuot DS, Davis E, Velasquez A, Banerjee T, Powe NR (2013). Assessment of printed patient-educational materials for chronic kidney disease. Am J Nephrol.

[34] Pickering TG, Hall JE, Appel LJ, Falkner BE, Graves J, Hill MN (2005). Recommendations for blood pressure measurement in humans and experimental animals: part 1: blood pressure measurement in humans: a statement for professionals from the subcommittee of professional and public education of the american heart association council on high blood pressure research. Circulation.

[35] Boulware LE, Troll MU, Jaar BG, Myers DI, Powe NR (2006). Identification and referral of patients with progressive CKD: a national study. Am J Kidney Dis.

[36] Campbell C, Lockyer J, Laidlaw T, Macleod H (2007). Assessment of a matched-pair instrument to examine doctor-patient communication skills in practising doctors. Med Educ.

[37] Ware J, Kosinski M, Keller SD (1996). A 12-item short-form health survey: construction of scales and preliminary tests of reliability and validity. Med Care.

[38] Morisky DE, Green LW, Levine DM (1986). Concurrent and predictive validity of a self-reported measure of medication adherence. Med Care.

[39] Lorig K, Stewart AF, Ritter P, Gonzalez V, Laurent D, Lynch J (1996). Outcome Measures for Health Education and Other Health Care Intervensions.

[40] Behavioral Risk Factor Surveillance System Survey Questionnaire. In. Atlanta, Ceorgia: The U.S. Department of Health and Human Services, Centers for Disease Control and Prevention (CDC); 2011.

[41] Glasgow RE, Nelson CC, Strycker LA, King DK (2006). Using RE-AIM metrics to evaluate diabetes self-management support interventions. Am J Prev Med.

[42] Carter BL, Rogers M, Daly J, Zheng S, James PA (2009). The potency of team-based care interventions for hypertension: a meta-analysis. Arch Intern Med.

[43] Inker LA, Astor BC, Fox CH, Isakova T, Lash JP, Peralta CA (2014). KDOQI US commentary on the 2012 KDIGO clinical practice guideline for the evaluation and management of CKD. Am J Kidney Dis.

[44] KDIGO (2012). Clinical practice guideline for the evaluation and management of chronic kidney disease. Kidney Int.

[45] Tuot DS, Plantinga LC, Hsu CY, Powe NR (2012). Is awareness of chronic kidney disease associated with evidence-based guideline-concordant outcomes?. Am J Nephrol.

[46] Tuot DS, Powe NR (2011). Chronic kidney disease in primary care: an opportunity for generalists. J Gen Intern Med.

[47] Tuot DS, Grubbs V (2015). Chronic kidney disease care in the US safety Net. Adv Chronic Kidney Dis.

[48] Willard-Grace R, Chen EH, Hessler D, DeVore D, Prado C, Bodenheimer T (2015). Health coaching by medical assistants to improve control of diabetes, hypertension, and hyperlipidemia in low-income patients: a randomized controlled trial. Ann Fam Med.

